# The Effects of Aging and Time of Day on Inhibitory Control: An Event-Related Potential Study

**DOI:** 10.3389/fnagi.2022.821043

**Published:** 2022-03-11

**Authors:** Rahel Rabi, Ricky Chow, Shahier Paracha, Lynn Hasher, Sandra Gardner, Nicole D. Anderson, Claude Alain

**Affiliations:** ^1^Baycrest Centre, Rotman Research Institute, Toronto, ON, Canada; ^2^Department of Psychology, University of Toronto, Toronto, ON, Canada; ^3^Biostatistics Division, Dalla Lana School of Public Health, University of Toronto, Toronto, ON, Canada; ^4^Department of Psychiatry, University of Toronto, Toronto, ON, Canada; ^5^Institute of Medical Science, University of Toronto, Toronto, ON, Canada

**Keywords:** aging, ERP, inhibitory control, chronotype, time of day, Go-NoGo task, Flanker task

## Abstract

Time of day (TOD) influences on executive functions have been widely reported, with greater efficiency demonstrated at optimal relative to non-optimal TOD according to one’s chronotype (i.e., synchrony effect). Older adults (OAs) show declines in inhibitory control and are more sensitive to the effects of circadian variation on executive functioning. To date, no studies have investigated the effects of TOD and aging on executive functioning using electrophysiological measures. The present study investigated the effects of aging and TOD on the neural correlates of inhibitory processing (N2 and P3) using event-related potentials (ERPs). Go-NoGo and Flanker tasks were administered to 52 OAs of morning chronotype and 51 younger adults (YAs) of afternoon-to-evening chronotype who were randomly assigned to morning or afternoon test sessions, with the optimal TOD for OAs in the morning and for YAs in the afternoon/evening. While behavioral results demonstrated no TOD effects, ERPs indicated synchrony effects. Both YAs and OAs showed greater modulation of Go-NoGo N2 and greater P3 amplitude during the non-optimal than optimal TOD, consistent with the synchrony effect. For the Flanker task, age differences in P3 amplitude were only apparent during the non-optimal TOD. These results suggest that processes associated with inhibitory control are differentially affected by TOD and aging, with age-related reductions in inhibitory efficiency during off-peak test times on measures of interference control. These findings highlight the sensitivity of ERPs to detect TOD effects in the absence of behavioral differences, confirm more pronounced TOD effects in OAs relative to YAs on ERP measures of interference control, and reinforce the need to assess and control for circadian typology in research studies.

## Introduction

Inhibitory control is a core component of executive functioning that underlies the ability to restrain inappropriate prepotent responses (i.e., response inhibition) and suppress irrelevant information (i.e., interference control; Zacks and Hasher, 1994; Diamond, 2013; Hasher and Campbell, 2020). This fundamental cognitive function is essential for coping with the demands of everyday life, such as avoiding distractions while driving or withholding the expression of socially inappropriate thoughts while engaging in conversation. Response inhibition refers to the ability to suppress an automatic or dominant response and is commonly measured by the Go-NoGo task (Go-NoGo, Mesulam, 1985). Interference control refers to the ability to filter out competing information present in the target or the environment, but irrelevant to the task being performed. It is commonly measured by the Erikson Flanker task (Eriksen and Eriksen, 1974). Among the different inhibition tasks used in prior behavioral studies, the Go-NoGo task and the Eriksen Flanker Task are widely paired with neuroelectric recording of event-related potentials (ERPs) to investigate response inhibition and interference control, respectively.

In the Go-NoGo task, participants must suppress an automatic or dominant response to infrequent items (i.e., NoGo trials) amidst a stream of standard items (i.e., Go trials). In the Flanker task, participants must ignore distractor arrowheads flanking a central arrowhead, with distractor arrowheads pointing either in a congruent or incongruent direction of the target. The electrophysiological correlates of inhibitory control most frequently observed in both tasks are the N2b and P3b waves (hereafter referred to as N2 and P3).

The N2 is a negative-going ERP deflection with a frontocentral scalp topography emerging 250–400 ms following stimulus onset (Kopp et al., 1996a,b; Heil et al., 2000; Liotti et al., 2000; Yeung et al., 2004; Bartholow et al., 2005). In general, the N2 represents early stage conflict monitoring processes that reflect conflict detection (Nieuwenhuis et al., 2003; Donkers and van Boxtel, 2004). In the Go-NoGo Task, the N2 amplitude has been found to be larger for NoGo than Go trials (Falkenstein et al., 1999; Van’t Ent and Apkarian, 1999; Folstein and Van Petten, 2008). In the Flanker task, the N2 has been found to be larger in amplitude and longer in latency for incongruent than congruent trials (Kopp et al., 1996a,b; van Veen and Carter, 2002; Frühholz et al., 2011; Brydges et al., 2012; Mansfield et al., 2013). Prior research has shown that response inhibition and interference control share similar early cognitive processes (Kan et al., 2021). As such, the N2 in Go-NoGo tasks reflects conflict arising from competition between the execution and inhibition of responses in Go vs. NoGo trials (Nieuwenhuis et al., 2003; Donkers and van Boxtel, 2004). In the Flanker task, it reflects conflict arising from distracting flankers surrounding the target (van Veen and Carter, 2002; Zhou et al., 2019). The P3 is a broad positive-going ERP waveform with a centroparietal scalp distribution that emerges approximately 300–600 ms following stimulus onset. More generally, the P3 is thought to reflect later monitoring and evaluation of inhibition processes and is closely related to actual inhibition of the motor response (Polich and Heine, 1996; Waller et al., 2021). Additionally, the P3 has been interpreted as indicating the effectiveness of the inhibitory response (Pfefferbaum et al., 1985; Roberts et al., 1994; Falkenstein et al., 1995). The P3 amplitude and latency are modulated by changing demands for inhibition and motor suppression, which have been shown to be task-specific. In the Go-NoGo task, P3 amplitudes have been found to be larger for NoGo than Go trials (Pfefferbaum et al., 1985; Bokura et al., 2001). In the Flanker task, the P3 has a smaller amplitude (Hsieh et al., 2012) and longer latency (Wild-Wall et al., 2008; Hsieh and Fang, 2012) for Incongruent than Congruent trials. Recent ERP research by Kan et al. (2021) demonstrated that response inhibition and interference control, assessed through a hybrid Go-NoGo Flanker task, share similar cognitive processes in the early stages (i.e., N2), but exhibit different temporal mechanisms in the later stages (i.e., P3) of inhibitory processing. More specifically, the Go-NoGo P3 reflects the actual process of response inhibition (i.e., active suppression of the motor response, Band and Van Boxtel, 1999; Kok et al., 2004) and the Flanker P3 reflects interference control and resolution needed to negotiate the conflict response demands of the target stimulus (Van ’t Ent, 2002; Scrivano and Kieffaber, 2022). According to the *Inhibitory Deficit Theory* (Hasher and Zacks, 1988; Hasher and Campbell, 2020; Amer et al., 2022), older adults (OAs) are less able to suppress or ignore irrelevant thoughts and actions relative to younger adults (YAs). Prior work has shown age-related behavioral deficits in response inhibition as indexed by increased reaction times (RTs) and error rates (Bedard et al., 2002; Bielak et al., 2006; Andrés et al., 2008; Potter and Grealy, 2008; Carriere et al., 2010; Hämmerer et al., 2010; but see Vallesi, 2011; Kardos et al., 2020). Additionally, past studies have found age-related deficits in interference control as indexed by larger RT and accuracy differences between congruent and incongruent trials (Zeef and Kok, 1993; Spieler et al., 1996; Zeef et al., 1996; Kok, 1999; West and Alain, 2000; van der Lubbe and Verleger, 2002; Proctor et al., 2005; Kawai et al., 2012; Chow et al., 2021; Scrivano and Kieffaber, 2022; but see Wild-Wall et al., 2008; Hsieh and Fang, 2012).

Electrophysiological studies investigating age-related differences in inhibitory control using the Go-NoGo task have shown differential N2 and P3 Go-NoGo effects between YAs and OAs, even in the absence of age differences in behavioral performance (Vallesi, 2011; Kardos et al., 2020). N2 amplitudes have been shown to be smaller in OAs than YAs, reflecting an age-related impairment of conflict-monitoring processes (Czigler et al., 1996; Lucci et al., 2013; Staub et al., 2014). With regards to P3 amplitude, a larger P3 modulation has been demonstrated in OAs relative to YAs (Vallesi et al., 2009; Vallesi and Stuss, 2010; Vallesi, 2011), suggesting that OAs experience increased difficulty in suppressing motor responses in No-Go trials and recruit more compensatory inhibitory mechanisms in suppressing irrelevant responses. Additionally, longer latencies of the No-Go N2 and No-Go P3 waves have been observed in OAs (Pfefferbaum and Ford, 1988; Tachibana et al., 1996; Falkenstein et al., 2002), reflecting general age-related slowing.

ERP studies involving the Flanker task have also supported the notion that OAs rely on compensatory strategies to inhibit irrelevant information to perform at a comparable level to YAs. Studies by Wild-Wall et al. (2008) and Hsieh and Fang (2012) did not find greater behavioral flanker effects in OAs relative to YAs. However, the ERP findings from both studies showed more pronounced between-condition modulations of the N2 in YAs than OAs and delayed P3 latencies in OAs compared to YAs. These results could indicate that OAs devote more attention to the central target to reduce flanker interference. Similar to the Go-NoGo task, larger P3 modulations in OAs relative to YAs have also been observed in the Flanker task (Korsch et al., 2016), suggesting that OAs use more inhibition resources to resolve conflicting information.

Findings from behavioral and electrophysiological studies provide support for the inhibitory deficit hypothesis of aging by demonstrating inhibitory differences in OAs relative to YAs. Evidence from behavioral studies on circadian rhythmicity have shown that these age-related differences in inhibitory control are modulated by time of day (TOD). That is, inhibitory control efficacy fluctuates across the day in synchrony with endogenous circadian rhythms. The *synchrony effect* refers to the interaction between chronotype and TOD, and involves better performance for optimal (i.e., morning time for morning-type individuals and evening time for evening-type individuals) as compared to non-optimal times of day (May and Hasher, 1998). Chronotype norms differ across age groups, with the majority of OAs being morning types and a substantial portion of YAs being or trending toward evening types (Mecacci et al., 1986; May et al., 1993; May and Hasher, 1998). This age-related shift in chronotype reflects a reliable developmental pattern where changes in morningness-eveningness preference co-varies with age-related changes in the internal body clock (Tankova et al., 1994; Roenneberg et al., 2007). These age trends in TOD preferences correspond with optimal performance for OAs during the morning, and for YAs during the evening. Synchrony effects have been demonstrated for both YAs and OAs in studies using behavioral measures of response inhibition and interference control (May et al., 1993, 1999; Intons-Peterson et al., 1998; May and Hasher, 1998; West et al., 2002; Borella et al., 2010; Knight and Mather, 2013; Anderson et al., 2014), with reduced inhibitory control abilities in both age groups during non-optimal relative to optimal times of day.

Inhibitory deficits in OAs have been found to be modulated by TOD in prior literature, with OAs demonstrating greater inhibition deficits in the evening relative to morning hours (May and Hasher, 1998; Borella et al., 2010; Anderson et al., 2014). Apart from an fMRI study by Anderson et al. (2014), research that has demonstrated TOD synchrony effects in OAs and YAs have relied on behavioral measures. No study to our knowledge has used electrophysiological measures to quantify TOD and aging influences on cognitive processes such as inhibitory control. The primary aim of this study was to investigate how TOD modulates behavioral and neural measures of two inhibitory control tasks between YAs and OAs.

In the current study, a Go-NoGo task was used to measure response inhibition and a Flanker task was used to measure interference control. To explore how TOD influences age-related differences in these two inhibitory control subtypes, participants performed inhibition tasks either during their optimal or non-optimal TOD (as established based on chronotype assessment). Therefore, we recruited YAs with evening chronotypes and OAs with morning chronotypes. Testing sessions were conducted in the morning or in the late afternoon to evening. Neuroelectric activity was recorded using electroencephalography (EEG) from both tasks to investigate how TOD influences the N2 and P3 inhibitory ERP components in both groups.

Our hypotheses combined existing behavioral and ERP research demonstrating inhibition deficits in aging (Vallesi et al., 2010; Vallesi, 2011; Hsieh and Fang, 2012; Kawai et al., 2012; Scrivano and Kieffaber, 2022) and behavioral and fMRI research demonstrating synchrony effects and TOD modulation of age-related differences in inhibitory efficiency (May and Hasher, 1998; Borella et al., 2010; Anderson et al., 2014). First, we expected to replicate prior aging work showing poorer response inhibition and interference control in OAs relative to YAs, as reflected by behavioral indices (i.e., larger reaction time and accuracy difference scores, calculated from the difference between a basic processing condition and an inhibition condition), and neural indices (i.e., larger modulation of ERP components indexing inhibition). Second, we expected a synchrony effect where both OAs and YAs perform worse and show larger N2 and P3 modulations during their non-optimal TOD relative to optimal TOD. Last, we used a novel ERP approach to studying TOD and aging effects on inhibitory control, expecting more pronounced age-related differences in inhibitory control, behaviorally and neurally during the non-optimal TOD, as OAs have been shown to be more susceptible to TOD effects than YAs.

## Materials and Methods

### Participants

Participants were recruited if they were native English speakers or learned English before the age of 5, had normal or corrected-to-normal vision (e.g., no history of degenerative conditions, glaucoma, cataracts significant enough to impede vision, or color blindness), and reported no significant hearing loss, no history of learning disabilities, stroke, transient ischemic attack, traumatic brain injury with loss of consciousness greater than 5 min, substance abuse disorder, neurodegenerative disease, brain abnormalities, history of intracranial surgery, and any other diagnosis of major neurological or psychiatric disorder. Participants were excluded if they had a history of myocardial infarction, coronary artery disease, or bypass surgery. Participants were also excluded if they were taking medications known to possibly affect cognitive functioning, including antidepressants, anticonvulsants, neuroleptics, or consuming recreational drugs either concurrently or within the year prior to testing. Inclusion criteria was scoring above the cut-off on the Telephone Interview for Cognitive Status-Modified (TICS-M, Welsh et al., 1993). Additionally, the Morningness-Eveningness Questionnaire (MEQ, Horne and Östberg, 1976) was used to ensure OA participants were of the morning chronotype, scoring at or above 59, and YA participants were of the evening chronotype, scoring at or below 46. The MEQ has been associated with circadian-related physiological changes such as body temperature, heart rate, and skin conductance (Horne and Östberg, 1976; Adan, 1991; Roenneberg et al., 2003).

Fifty-five YAs and 54 OAs were recruited for the study. One YA and one OA did not complete the inhibition tasks. Data from two YAs were excluded due to impaired neuropsychological performance. One YA and one OA reported inadequate sleep the night before testing, and their data were also excluded.

Our final sample consisted of 51 YAs (18–30 years, 25 females), and 52 OAs (64–88 years, 25 females). Participants were randomly assigned to perform the inhibition tasks during optimal or non-optimal TOD. Individuals were recruited from the Rotman Research Institute research participant pool, and through local advertisements and community talks. YAs and OAs did not statistically differ in gender, *X*^2^(1, *N* = 103) = 0.009, *p* = 0.924. Additionally, gender did not statistically differ between participants assigned to optimal and non-optimal testing times within YAs, *X*^2^(1, *N* = 51) = 0.174, *p* = 0.676, or OAs, *X*^2^ (1, *N* = 52) = 0.077, *p* = 0.781. YAs and OAs statistically differed in education level, *t*(101) = –3.006, *p* = 0.003. OAs (*M* = 16.50) were significantly more educated than YAs (*M* = 15.06), which is expected as many of the YAs in our study had not completed their schooling yet. Importantly, years of education did not statistically differ between participants assigned to optimal and non-optimal testing times within YAs, *t*(49) = –0.978, *p* = 0.333, or OAs, *t*(50) = –1.590, *p* = 0.118. Finally, YAs in the optimal vs. non-optimal TOD groups did not differ in Eveningness on the MEQ, *t*(49) = 0.322, *p* = 0.749 and OAs in the optimal vs. non-optimal TOD groups did not differ in Morningness on the MEQ, *t*(50) = 0.235, *p* = 0.815. The study protocol was approved by the Research Ethics Board of the Rotman Research Institute at Baycrest Centre. Informed written consent was obtained from all participants.

### Neuropsychological Assessment

OA participants were administered a battery of neuropsychological tests in the domains of intellectual functioning, memory, language, processing speed, and executive functioning. YA participants were administered an abbreviated version of the neuropsychological battery. To ensure optimal cognitive performance, all neuropsychological assessments took place during their optimal TOD (morning for OAs, or 9:00–12:00; afternoon for YAs, or 14:00–18:30). For OAs, the Montreal Cognitive Assessment (MoCA; Nasreddine et al., 2005) was administered to assess global cognitive ability. The Shipley’s Institute of Living Scale II (SILS-II, Shipley et al., 2009) was administered to estimate crystalized intelligence, and the Wechsler Adult Intelligence Scale Matrix Reasoning (WAIS-MR, Wechsler, 1997) to estimate fluid intelligence. Processing speed was assessed with the WAIS Digit Symbol Coding subtest (WAIS-III DS, Wechsler, 1997), the Delis-Kaplan Executive Function System Trail Making Test (Number and Letter subtests) (D-KEFS TMT, Delis et al., 2001), and D-KEFS Color-Word Interference Test (Color Naming and Word Reading subtests) (D-KEFS CWIT, Delis et al., 2001). Memory was assessed using the California Verbal Learning Test II (CVLT-II, Delis et al., 2000), Incidental and Free Recall subsections of the Digit Symbol Coding Test, Verbal Paired Associates, and Visual Paired Associates subtests from the Wechsler Memory Scale—Revised (WMS-R Visual and Verbal Paired Associates, Wechsler, 1987). To assess phonemic and semantic fluency, the F-A-S and Animal Fluency Tests (Spreen and Strauss, 1998) were administered, while the short form of the Boston Naming Test (BNT-SF; Kaplan et al., 1983) was used as a naming measure. Executive functioning measures included the Wisconsin Card Sorting Test (WCST, Grant and Berg, 1948), Alpha Span Test (Craik et al., 2018), the D-KEFS TMT, Number-Letter Switching Subtest (Delis et al., 2001), and D-KEFS CWIT Inhibition subtest (Delis et al., 2001). YAs completed only the SILS-II, WAIS-MR, and the Incidental Learning Paired Recall and Free Recall subtests of the WAIS-III DS.

Only OAs completed additional questionnaires. The Epworth Sleepiness Scale (Johns, 2000) and Pittsburgh Sleep Quality Index (Buysse et al., 1989) were administered to assess sleep quality. The memory assessment clinics self-rating scale (Feher et al., 1994) was administered to assess subjective memory concern. Self-reported functional independence was assessed with the Basic Activities of Daily Living Scale and the Instrumental Activities of Daily Living Scale (Lawton and Brody, 1969), and verified through the Functional Assessment Questionnaire (Pfeffer et al., 1982) by a reliable third-party informant. Both YAs and OAs completed the Hospital Anxiety and Depression Scale (Zigmond and Snaith, 1983).

### Procedure

All OAs performed the inhibition tasks on a separate day than the neuropsychological assessment. OAs in the Optimal TOD and YAs in the Non-Optimal TOD completed the inhibition tasks in the morning (8:00–10:30 start time, with all tasks completed by 12:00); YAs in the Optimal TOD and OAs in the Non-Optimal TOD completed the inhibition tasks in the late afternoon to evening (14:00–17:00 start time, with all tasks completed by 18:30). The order of inhibition tasks was counterbalanced across participants, and no participant was familiar with either task. All inhibition tasks were performed seated in a sound-attenuated booth 60 cm in front of a computer monitor with a visual angle of 2.9 degrees for the Go-NoGo task, and 3.8 degrees for the Flanker task.

### Computer Tasks

**FIGURE 1:**
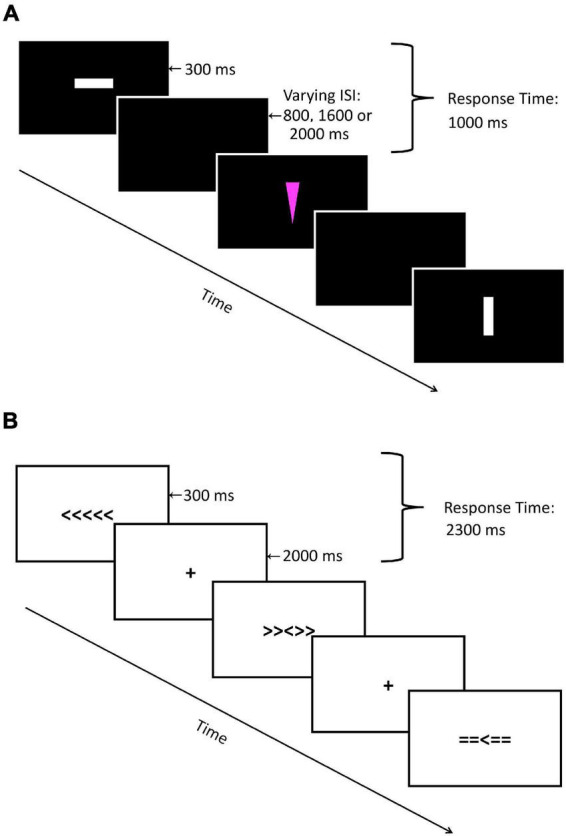
Visual representation of **(A)** Go-NoGo Task and **(B)** Flanker Task.

For the present study, we adopted the Go-NoGo paradigm used by Moussard et al. (2016). Geometrical shapes were presented on a computer monitor. There were four stimuli created from two shapes (triangles or squares) in two different colors (white or pink) to reduce stimulus repetition effects. Figure 1A depicts the sequence of events for each trial. A colored shape was presented on a black background for 186 ms followed by a fixed blank screen interstimulus interval lasting 1,500, 2,000, or 2,500 ms to prevent expectancy effects. Stimulus color assignment to standard or infrequent stimuli was counterbalanced across participants to control for stimulus saliency. Participants were instructed to press the spacebar on a regular computer keyboard in response to standard shapes (Go trials) as quickly and accurately as possible (75% probability) and to withhold responding to infrequent shapes (NoGo trials; 25% probability). The response time window was 1,000 ms from the onset of the stimulus. The paradigm consisted of 576 trials (432 Go and 144 NoGo trials) in total, separated into three blocks of 192 trials each. A practice block of 20 trials was used to familiarize participants with the task.

For the Flanker task, arrays of arrowheads were presented on a computer monitor. There were three different stimuli arrays, each with five symbols centered horizontally on the monitor, with each array comprising a centered arrowhead pointing to either left or right, and two flanker symbols on either side. Congruent arrays consisted of five arrowheads pointing in the same direction (e.g., > > > > >). Incongruent arrays consisted of four flanking arrowheads pointing in the direction opposite of the central arrowhead (e.g., > > < > >). Neutral arrays consisted of a middle arrowhead flanked by four equal signs (e.g., = = > = =). Figure 1B displays the sequence of events for each trial. A stimulus array was presented on a white background for 300 ms followed by a fixed interstimulus interval of 2,000 ms with a central fixation cross. Participants were instructed to press an arrow key on a standard keyboard in response to the direction of the central arrowhead as quickly and accurately as possible. Participants used the left index finger to respond to the centered arrowhead facing left, and the right index finger for the centered arrowheads facing right. The response time window was 2,300 ms from stimulus onset. The paradigm consisted of 306 trials in total (102 trials per condition) separated into three blocks of 102 trials each, with trial order randomized across participants. A practice block of 17 trials was used to familiarize participants with the task.

Stimuli for both tasks were displayed using E-Prime version 1.2 (Psychology Software Tools, Inc.). During the tasks, participants did not receive any feedback on their performance.

### Electroencephalography Acquisition and Preprocessing

Neuroelectric brain activity was recorded from 66 Ag/AgCl scalp electrodes using a BioSemi Active Two acquisition system (BioSemi V.O.F., Amsterdam, Netherlands). The electrode montage included electrodes from the 10/20 system, a common mode sense active electrode and driven right leg passive electrode serving as ground. Ten additional facial electrodes were added, which were placed below the hair line (both mastoid, both pre-auricular points, outer canthus of each eye, inferior orbit of each eye, and two additional frontotemporal electrodes) to monitor eye movements and to cover the whole scalp evenly. Neuro-electric activity was digitized continuously at a rate of 512 Hz with a bandpass of DC-100 Hz, and stored for offline analysis. All off-line preprocessing was performed using Brain Electrical Source Analysis software (BESA Research, version 7.0, MEGIS GmbH, Gräfelfing, Germany).

For ERP analysis, an average reference (i.e., the average of all scalp EEG channels as the reference for each EEG channel) was used. Continuous EEG data were digitally filtered with 0.53 Hz high-pass (forward, 6 dB/octave) and 40 Hz low-pass filters (zero phase, 24 dB/octave). These filter settings were chosen for two reasons. The first reason was to attenuate slow-wave potentials that may not be specific to the actual response suppression and cognitive control processes. Second, given that our older adult participants were prone to head movements, we adopted a higher cut-off frequency of 0.53 Hz.

Channels with excessive artifacts, such as those caused by head or body movements, were then interpolated using spherical spline interpolation (Picton et al., 2000), with no more than 10% of the channels per recording interpolated. For each participant, artifacts from ocular movements were corrected from the continuous EEG recording based on the spatial components approach (Berg and Scherg, 1994). Brain signal topographies underlying lateral and horizontal eye movements, as well as eyeblinks, were semi-automatically detected per participant recording, then the artifact signal for each electrode was reconstructed with a spatial filter and modeled by a fixed dipole model (Berg and Scherg, 1994). The spatial topographies were then subtracted from the continuous EEG.

After correcting for eye movements, data for each participant were then segmented into epochs of –500 to 1,000 ms with a baseline of –500 to 0 ms. Only correct trials were included in the ERP analysis. Epochs were scanned for additional artifacts, with epochs including deflections exceeding a 60 μV difference between the maximum and the minimum amplitude for a given epoch and channel marked and excluded from the analysis. This removed an average of 6.52% (*SD* = 5.53%) of trials per participant in the Go-NoGo task and 4.56% (*SD* = 4.45%) of trials per participant in the Flanker task, neither of which varied by age group, *F*(1, 101) = 3.34, *p* = 0.059, η*_*p*_*^2^ = 0.035, and *F*(1, 101) = 2.64, *p* = 0.107, η*_*p*_*^2^ = 0.025, respectively. The remaining epochs were averaged according to experimental conditions, and averaged epochs were baseline-corrected with respect to the pre-stimulus interval (i.e., mean amplitude over the 500 ms prior to stimulus onset). Waveforms for the Go-NoGo task included a mean of 404 Go trials (*SD* = 37.55) and 120.03 NoGo trials (*SD* = 14.70) per participant; for the Flanker task, these were 97.02 (*SD* = 7.91) for Congruent, 86.66 (*SD* = 11.27) for Incongruent, and 96.35 *(SD* = 8.43) for Neutral conditions.

### Data Preparation

#### Behavioral Measures

Go-NoGo mean accuracy values were calculated from both hits and correct rejections. Go-NoGo mean RT values were calculated only from hits (Go condition). For the Flanker task, trials without a response were discarded from accuracy calculations. The first trial in each block was omitted to accommodate for task warm-up effects. Trials with a response time less than 200 ms were also removed. Mean accuracy for the Flanker task was calculated for the Congruent, Incongruent and Neutral conditions. Mean RT was calculated using correct trials and any trials with an RT 3 standard deviations or more from the participants’ mean in each condition were removed. This removed on average 1.36% (*SD* = 0.64%) of Go trials per participant in the Go-NoGo task, with significantly more Go trials trimmed in OAs (1.57%, *SD* = 0.64%) than YAs (1.15%, *SD* = 0.58%), *F*(1, 101) = 11.75, *p* = 0.001, η*_*p*_*^2^ = 0.104. In the Flanker task, this removed 1.41% (*SD* = 0.65%) of trials per participant, which did not vary by age group, *F*(1, 101) = 1.34, *p* = 0.250, η*_*p*_*^2^ = 0.013. No participant scored significantly below chance within a condition for a given block of trials (below 29% correct within a single condition in the Flanker task, below 19% correct within a single condition in the Go-NoGo task).

#### Event-Related Potential Measures

Peak latencies were measured as the maximum positivity or negativity within a specific time window averaged over a predefined electrode cluster. The N2 modulation for both tasks was maximal at frontal-central regions, and therefore latencies and mean amplitude were localized and averaged across a cluster of nine electrodes in the frontal-central scalp region (F1, Fz, F2, FC1, FCz, FC2, C1, Cz, C2). The Go-NoGo P3 was maximal at central scalp regions, and was therefore averaged across a cluster of nine central electrodes (FC1, FCz, FC2, C1, Cz, C2, CP1, CPz, CP2); the Flanker P3 modulation was maximal at centro-parietal regions, and therefore latencies and mean amplitude were averaged across a cluster of nine central-parietal electrodes (C1, Cz, C2, CP1, CPz, CP2, P1, Pz, P2).

Go-NoGo and Flanker N2 peak latencies were exported from each participant at the latency of the maximal negative-going peak between a time window of 200–400 ms for both groups.^1^ Go-NoGo and Flanker P3 peak latencies were measured for each participant at the maximal positive-going peak between 250–650 ms for both age groups.

Upon visual inspection of grand mean waveforms, the N2 and P3 for the Go-NoGo task and the P3 from the Flanker task showed longer peak latencies in OAs than YAs, and so mean amplitudes for these measures were extracted with different time windows between age groups. Mean amplitudes for the Go-NoGo N2 were derived from a time window of 230–330 ms for YAs and 250–350 ms for OAs. For the Go-NoGo P3, YAs mean amplitudes were derived using a time window of 300–550 ms, and for OAs, 350–600 ms. Flanker N2 mean amplitudes were derived from the same time window of 225–350 ms for both groups due to similar grand peak latencies for both age groups. Flanker P3 mean amplitudes were derived from a 250 ms long time window of 250–550 ms for YAs and 350–600 ms for OAs.

### Data Analysis

#### Behavioral Measures

Statistical analysis of behavioral data was performed using SAS/STAT software version 15.2 and the SAS System for Windows version 9.4. Copyright© 2016 SAS Institute Inc. To accommodate the positively skewed distribution of RT, generalized linear mixed models (GLMMs) for gamma distribution (with an identity link) were used to model the individual trial RT data (Lo and Andrews, 2015). A random intercept and a variance component structure were included to control for the non-independence of the data (i.e., repeated measures within subject).

For accuracy data, the dependent variable was a dichotomous variable (0 = error; 1 = correct response) and the data were fit to a modified Poisson model (Zou, 2004). This model estimates the proportion correct and ratios of proportions correct across groups. The model used generalized estimating equations and a compound symmetry type working correlation matrix to adjust for the repeated measures within subject.

For each outcome, the initial model contained the interaction of all fixed effects. If the 3-way interaction, for example, was not significant, a model with only 2-way interactions was considered. The model was simplified to contain only significant interactions and related main effects or only main effects when no interaction terms were significant (see Supplementary Tables 1, 2 for the models used for each analysis for the different tasks). The following fixed effects were included: Group (YAs, OAs), TOD (Optimal, Non-Optimal) and condition (Go-NoGo Task: Go, No-Go; Flanker Task: Incongruent, Neutral, Congruent).

For RTs, an initial gamma GLMM model was fit without any fixed effects (an intercept model only). The standard deviation of the predicted mean RT was used as the denominator of effect size calculations for differences in mean RT across groups. Effect sizes were interpreted according to Cohen’s d criteria (Cohen, 1988), where an effect size of 0.2 indicates a small effect, 0.5 a medium effect, and > 0.8 a large effect.

#### Event-Related Potential Measures

ERP analyses were conducted using IBM SPSS (version 28.0) and JASP (version 0.16) software. Electrophysiological measures were subjected to mixed model ANOVAs conducted with Sidak correction to compensate for multiple comparisons. Partial eta-squared was calculated as a measure of effect size. A Greenhouse-Geisser correction was used when the assumption of sphericity had been violated. An alpha value of 0.05 was used throughout. Analyses focused on the difference wave (e.g., “NoGo minus Go” or “Incongruent minus Congruent or Neutral”) for the N2 and P3 waves, which have shown to more clearly index inhibitory control performance relative to examining each condition in the inhibition task separately (Salthouse and Meinz, 1995; Moussard et al., 2016).

Mean amplitudes and peak latencies for the N2 and P3 components were subjected to mixed model analyses of variance (ANOVA) with Group (YAs, OAs) and TOD (Optimal, Non-Optimal) as between-subjects factors, and Condition (Incongruent, Neutral, Congruent for Flanker; Go and NoGo for Go-NoGo) as the within-subjects factor. For significant three-way interactions in the Flanker task, follow-up two-way ANOVAs were run for significant interactions involving Condition: N2 and P3 amplitude modulations for Incongruent-Congruent and Incongruent-Neutral Flanker effects.

For each age group, bivariate Pearson correlation analyses were performed between ERP and behavioral measures of inhibitory control to confirm that N2 and P3 modulations were associated with inhibition in OAs and YAs.^2^ As amplitude modulations were taken as a measure of the Go-NoGo or Flanker effect and reflect inhibitory processing (i.e., the difference between Go and NoGo, or between Incongruent and Congruent/Neutral), correlations were run between N2 or P3 modulations and inhibition performance (i.e., Go-NoGo or Flanker effect in accuracy and RT). As peak latencies were taken as a measure of age- or TOD-related slowing of neural processing and did not index inhibitory control, correlations were not run between N2 or P3 latencies and accuracy or RT. Fisher’s *z*-test was used to compare correlations between age groups.

## Results

### Participant Demographics

Data from the neuropsychological assessment are summarized in Table 1. Consistent with previous studies, we observed the typical age-related increase in crystallized intelligence with OAs having higher vocabulary scores than YAs. In the WAIS Digit Symbol Free Recall test, we observed a typical age-related decrease in incidental recall with OAs performing worse than YAs (Voelcker-Rehage and Alberts, 2007; Scheibe and Blanchard-Fields, 2009).

**TABLE 1:** Participant characteristics and neuropsychological test scores.

Variable	YA AM Mean (SD) *(n = 26)*		YA PM Mean (SD) *(n = 25)*		OA AM Mean (SD) *(n = 26)*		OA PM Mean (SD) *(n = 26)*	
	
	Raw	Scaled	Raw	Scaled	Raw	Scaled	Raw	Scaled
**Demographics**								
Age (years)	20.96 (2.18)	–	22.12 (3.38)	–	75.15 (7.38)	–	75.23 (5.40)	–
Education (years)	14.83 (1.83)	–	15.36 (2.06)	–	15.88 (3.01)	–	17.12 (2.55)	–
Gender (F:M)	12:14	–	13:12	–	12:14	–	13:13	–
TICS-M	38.24 (3.17)	–	37.80 (3.89)	–	37.42 (2.76)	–	36.96 (3.29)	–
MEQ*	41.00 (6.13)	–	40.52 (4.32)	–	65.96 (4.79)	–	65.65 (4.66)	–
MoCA	–	–	–	–	26.92 (2.42)	–	26.88 (2.39)	–
**Estimates of IQ**								
WAIS-III Matrix Reasoning	28.23 (3.41)	15.04 (20.30)	29.00 (3.67)	11.72 (2.37)	24.46 (3.80)	14.54 (2.23)	24.04 (5.59)	14.31 (2.62)
Shipley Vocabulary*	31.58 (3.96)	11.81 (2.06)	30.29 (4.32)	10.92 (2.21)	35.46 (2.98)	12.00 (2.24)	36.19 (3.68)	12.96 (3.14)
**Memory**								
CVLT-II Learning	–	–	–	–	49.28 (9.06)	13.08 (2.29)	50.12 (14.63)	13.77 (3.29)
CVLT-II Short Delay FR	–	–	–	–	10.52 (3.33)	12.14 (3.03)	10.27 (3.56)	12.12 (3.15)
CVLT-II Long Delay FR	–	–	–	–	10.40 (3.32)	11.36 (2.86)	10.92 (3.62)	11.85 (2.89)
WMS-R Visual PA I	–	–	–	–	12.36 (3.34)	12.00 (2.66)	11.92 (3.58)	11.81 (2.53)
WMS-R Visual PA II	–	–	–	–	5.04 (1.40)	11.84 (1.75)	5.12 (1.37)	12.15 (1.46)
WMS-R Verbal PA I	–	–	–	–	15.72 (3.37)	9.60 (2.24)	17.11 (2.98)	10.88 (2.44)
WMS-R Verbal PA II	–	–	–	–	6.92 (1.04)	11.84 (1.84)	7.00 (1.17)	12.00 (2.47)
WAIS-III Digit Symbol IL-FR*	8.08 (1.20)	9.69 (1.91)	8.12 (1.01)	8.62 (1.50)	7.58 (1.06)	10.54 (0.99)	7.42 (1.21)	10.38 (1.33)
WAIS-III Digit Symbol IL-PR	15.35 (3.08)	10.35 (1.09)	15.32 (3.70)	10.24 (1.45)	12.58 (4.37)	10.50 (1.36)	12.73 (4.37)	10.65 (1.02)
**Language**								
BNT-15	–	–	–	–	53.60 (5.77)	10.80 (3.33)	54.08 (3.77)	11.12 (2.70)
Phonemic Fluency (FAS)	–	–	–	–	48.58 (13.31)	12.00 (3.00)	49.88 (13.17)	12.04 (3.56)
Semantic Fluency (Animal)	–	–	–	–	17.54 (4.45)	9.81 (2.80)	19.46 (5.09)	10.81 (3.70)
**Executive functioning and processing speed**								
WAIS-III Digit Symbol	95.42 (16.74)	12.81 (3.33)	89.72 (17.75)	12.00 (3.30)	57.96 (13.82)	12.23 (2.80)	65.58 (14.75)	13.69 (2.99)
D-KEFS Trails Numbers	–	–	–	–	37.87 (10.72)	12.92 (2.10)	39.27 (15.45)	12.88 (2.41)
D-KEFS Trails Letters	–	–	–	–	36.56 (10.06)	12.96 (1.43)	40.49 (13.89)	12.63 (1.84)
D-KEFS Trails N-L Switch	–	–	–	–	95.13 (36.85)	12.25 (2.13)	94.51 (44.80)	12.35 (2.81)
D-KEFS CWIT Color	–	–	–	–	30.31 (5.89)	11.58 (2.22)	30.88 (4.85)	11.42 (1.93)
D-KEFS CWIT Word	–	–	–	–	23.51 (5.11)	11.21 (2.64)	22.31 (4.77)	11.88 (2.36)
D-KEFS CWIT Inhibition	–	–	–	–	58.61 (15.17)	12.88 (2.07)	56.78 (9.17)	13.08 (1.35)
Alpha Span	–	–	–	–	27.68 (9.58)	10.12 (2.99)	30.04 (11.97)	11.08 (3.67)
WCST Categories	–	–	–	–	4.81 (1.96)	–	5.00 (1.62)	–
WCST Perseverative Errors%	–	–	–	–	14.35 (10.04)	13.52 (4.74)	14.15 (10.28)	12.92 (4.53)
**Questionnaires**								
HADS Anxiety*	6.35 (3.93)	–	6.00 (3.51)	–	5.08 (3.31)	–	4.08 (2.33)	–
HADS Depression*	3.52 (2.50)	–	3.40 (2.16)	–	2.12 (1.90)	–	2.70 (2.00)	–
EPW	–	–	–	–	6.60 (2.89)	–	7.50 (3.18)	–
PSQI	–	–	–	–	5.75 (3.43)	–	6.16 (2.53)	–

*YA, younger adult; OA, older adult; MoCA, Montreal Cognitive Assessment; TICS-M, Modified Telephone Interview of Cognitive Status (raw score out of 50); MEQ, Morningness-Eveningness Questionnaire; WAIS, Wechsler Adult Intelligence Scale; WMS-R, Wechsler Memory Scale—Revised; CVLT, California Verbal Learning Test; FR, Free Recall; PA, Paired Associates; IL, Incidental Learning; PR, Paired Recall; BNT, Boston Naming Test; FAS, phonemic fluency to the letters F, A, and S; D-KEFS, Delis Kaplan Executive Functioning System; N-L, Number-Letter; CWIT, Color Word Interference Test; WCST, Wisconsin Card Sorting Test; HADS, Hospital Anxiety and Depression Scale; EPW, Epworth Sleepiness Scale; PSQI, Pittsburgh Sleep Quality Index.*

*YA≠OA, p < 0.05.

### Behavioral Results

#### Go-NoGo Performance

**FIGURE 2:**
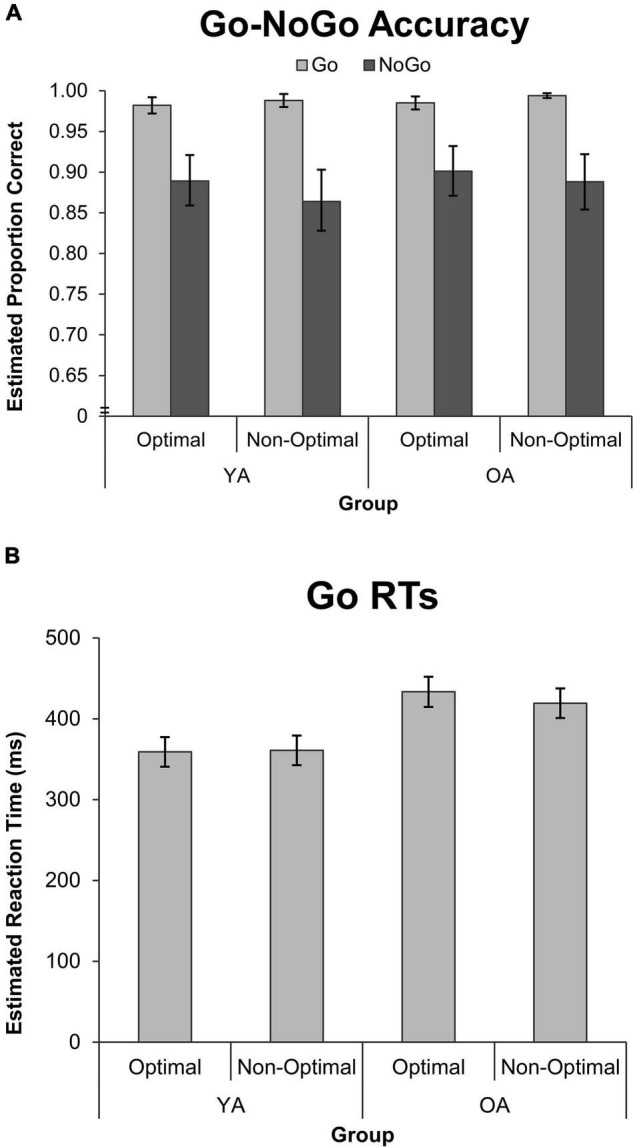
Performance on the Go-NoGo Task by group, TOD, and condition for measures of **(A)** GLMM estimated accuracy, and **(B)** GLMM estimated mean RT. Error bars represent the 95% confidence interval, and the *y*-axis scale for accuracy is truncated to aid in visualizing the Go-NoGo effect. OA, older adults; YA, younger adults; RT, reaction time.

Go-NoGo accuracy did not differ between groups or as a function of TOD (see Supplementary Table 1), and so the model was simplified to contain only the Condition main effect. As depicted in Figure 2, participants made more mistakes in NoGo than Go trials, χ^2^(1) = 57.46, *p* < 0.001, *accuracy ratio* = 0.897, indicating a ∼11% decrease in NoGo accuracy relative to Go accuracy across groups.

Go RTs were not modulated by TOD (see Supplementary Table 1), so the model was simplified to just examine the Group main effect. As expected, OAs had longer Go RTs than YAs, *F*(1, 43,329) = 50.35, *p* < 0.001, *effect size* = 0.570.

#### Flanker Performance

**FIGURE 3:**
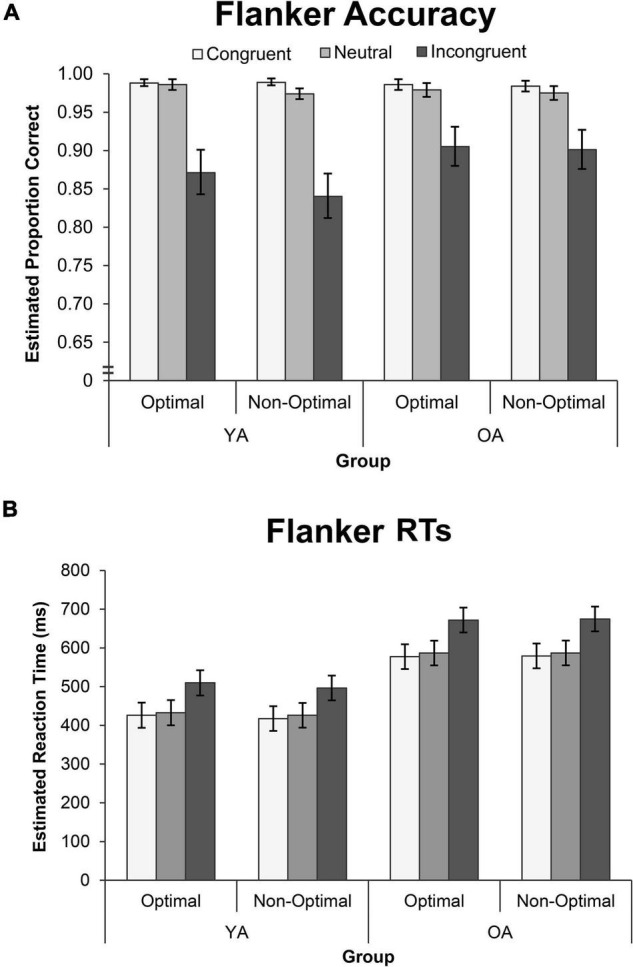
Performance on the Flanker Task by group, TOD and condition for measures of **(A)** GLMM estimated accuracy, and **(B)** GLMM estimated mean RT. Error bars represent the 95% confidence interval. OA, older adults; YA, younger adults; RT, reaction time.

Flanker behavioral results are displayed in Figure 3. TOD did not influence accuracy on the Flanker task (see Supplementary Table 2), and so the model was simplified to Group, Condition, and their interaction. Accuracy was comparable for YAs and OAs χ^2^(1) = 2.73, *p* = 0.099, and differed across Condition, χ^2^(2) = 58.32, *p* < 0.001, but these effects interacted, χ^2^(2) = 7.68, *p* = 0.022. Pairwise contrasts showed that OAs had a 6% increase in accuracy in the Incongruent condition, compared to both the Congruent condition (*accuracy ratio* = 1.060, *p* = 0.006) and the Neutral condition (*accuracy ratio* = 1.059, *p* = 0.004), relative to YAs.

Flanker RTs were not modulated by TOD for either group, or in any condition (see Supplementary Table 2), and so the model was simplified to Group, Condition, and their interaction. RTs were longer for OAs than YAs, *F*(1, 29,125) = 99.33, *p* < 0.001, and differed across conditions, *F*(1, 29,125) = 3583.02, *p* < 0.001, but these effects interacted. The Group by Condition interaction was significant, *F*(1, 29,125) = 22.09, *p* < 0.001. Pairwise contrasts showed that RTs in the Incongruent condition were longer for OAs than YAs, relative to both the Congruent condition (*effect size*_inc–con_ = 0.095) and Neutral condition (*effect size*_inc–neu_ = 0.088).

To summarize, TOD did not modulate inhibition performance, nor was the age difference in inhibitory control modulated by TOD. Accuracy was quite high in the control conditions across tasks (i.e., Go trials in the Go-NoGo task; Congruent and Neutral trials in the Flanker task). Such ceiling effects could have limited our ability to detect differences across TOD. Furthermore, age differences were significant only for Go RTs and in the Flanker effect, although the effect sizes of the latter were negligible, for both accuracy and RT metrics.

### Go-NoGo Event-Related Potential Results

A summary of statistics for ERP data and N2 and P3 peak latencies and mean amplitudes for the Go-NoGo task is displayed in Supplementary Tables 3, 4.

**FIGURE 4:**
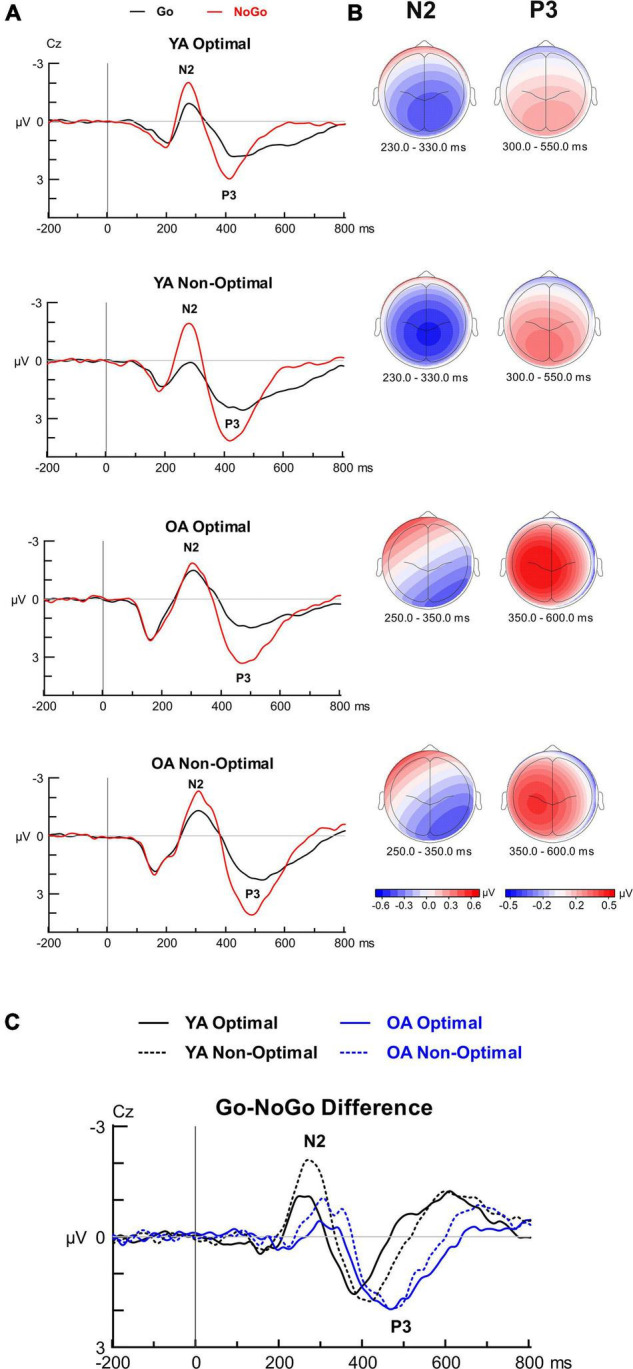
Grand average waveforms, scalp topographies and difference waveforms at electrode site Cz for the Go-NoGo task. **(A)** Grand-average ERPs time-locked to stimulus onset and averaged over go and nogo conditions separately for the four groups depicting N2 and P3 components. **(B)** Topographical iso-contour maps and **(C)** difference waveforms (NoGo—Go) depicting the N2 and P3 components for the four groups.

**N2.** Figure 4 displays the Go-NoGo ERP findings. For N2 peak latency, there were no significant interactions involving Group, Condition, or TOD. Overall, the N2 latencies were longer in OAs than YAs, *F*(1, 99) = 19.49, *p* < 0.001, η*_*p*_^2^* = 0.165. The analysis for Go-NoGo N2 mean amplitudes showed a Group by Condition interaction, *F*(1, 99) = 12.45, *p* = 0.001, η*_*p*_^2^* = 0.112, with a greater N2 modulation (i.e., greater N2 amplitude difference between conditions) in YAs than OAs. Pairwise comparisons showed, as expected, greater N2 amplitudes for NoGo than Go trials in YAs (*p* < 0.001), whereas N2 amplitude did not differ between conditions in OAs (*p* = 0.594). Additional pairwise comparisons showed greater NoGo N2 amplitudes in YAs than OAs (*p* < 0.001), whereas Go N2 amplitudes did not significantly differ between groups (*p* = 0.121). The analysis also showed a Condition by TOD interaction, *F*(1, 99) = 6.96, *p* = 0.010, η*_*p*_*^2^ = 0.066, with a greater N2 amplitude modulation for the Non-Optimal than Optimal TOD. In the Optimal TOD, N2 amplitudes were larger for NoGo than Go (*p* < 0.001); in the Non-Optimal TOD, N2 amplitudes were not significantly different between conditions (*p* = 0.923). No other effects or interactions involving Group, Condition, or TOD reached significance for N2 amplitude. ERP-behavior correlations between the N2 modulation and the Go-NoGo effect in accuracy did not reach significance for either YAs (*r* = 0.155, *p* = 0.277) or OAs (*r* = 0.067, *p* = 0.636), and there was no significant difference between correlations (*z* = 0.044, *p* = 0.330).

**P3.** For P3 peak latency, there were no significant interactions involving Group, Condition, or TOD. However, Go-NoGo P3 latencies were longer in OAs than YAs, *F*(1, 99) = 14.54, *p* < 0.001, η*_*p*_^2^* = 0.128. P3 latencies were also longer in Go than NoGo trials, *F*(1, 99) = 10.98, *p* = 0.001, η*_*p*_^2^* = 0.100. The analysis for Go-NoGo P3 amplitude showed a Group by Condition interaction, *F*(1, 99) = 45.90, *p* = 0.0006, η*_*p*_*^2^ = 0.075, with a significantly greater P3 amplitude modulation in OAs than YAs. Simple effects analyses by Group showed larger P3 amplitudes for NoGo than Go trials in OAs (*p* < 0.001). Additional simple effects analyses by Condition showed greater NoGo P3 amplitudes in OAs than YAs (*p* = 0.022), whereas Go P3 amplitudes did not significantly differ between group (*p* = 0.894). A main effect of TOD was also shown, *F*(1, 99) = 4.37, *p* = 0.039, η*_*p*_*^2^ = 0.042, with larger P3 amplitudes for the Non-Optimal than Optimal TOD. No other effects or interactions involving Group, Condition, or TOD reached significance for P3 latency or amplitude. ERP-behavior correlations demonstrated that, in YAs, a greater P3 amplitude modulation was significantly correlated with a greater Go-NoGo effect in accuracy (*r* = 0.423, *p* = 0.002), whereas this correlation did not reach significance in OAs (*r* = 0.231, *p* = 0.099). There was a significant difference in correlations between age groups (*z* = –1.06, *p* = 0.144).

**Summary:** OAs demonstrated longer N2 and P3 latencies than YAs in both Go and NoGo conditions, as expected from prior research (Vallesi, 2011; Mudar et al., 2015). Additionally, we found evidence supporting synchrony effects with YAs and OAs showing a greater Go-NoGo N2 amplitude modulation and greater P3 amplitude for non-optimal than optimal testing times. YAs also showed greater Go-NoGo N2 amplitude modulations than OAs. As expected, OAs demonstrated a greater Go-NoGo P3 amplitude modulation than YAs which did not change with TOD; this P3 modulation tended to index inhibition performance.

### Flanker Event-Related Potential Results

A summary of statistics for ERP data, as well as N2 and P3 peak latencies and mean amplitudes for the Flanker task are displayed in Supplementary Tables 3, 4.

**FIGURE 5:**
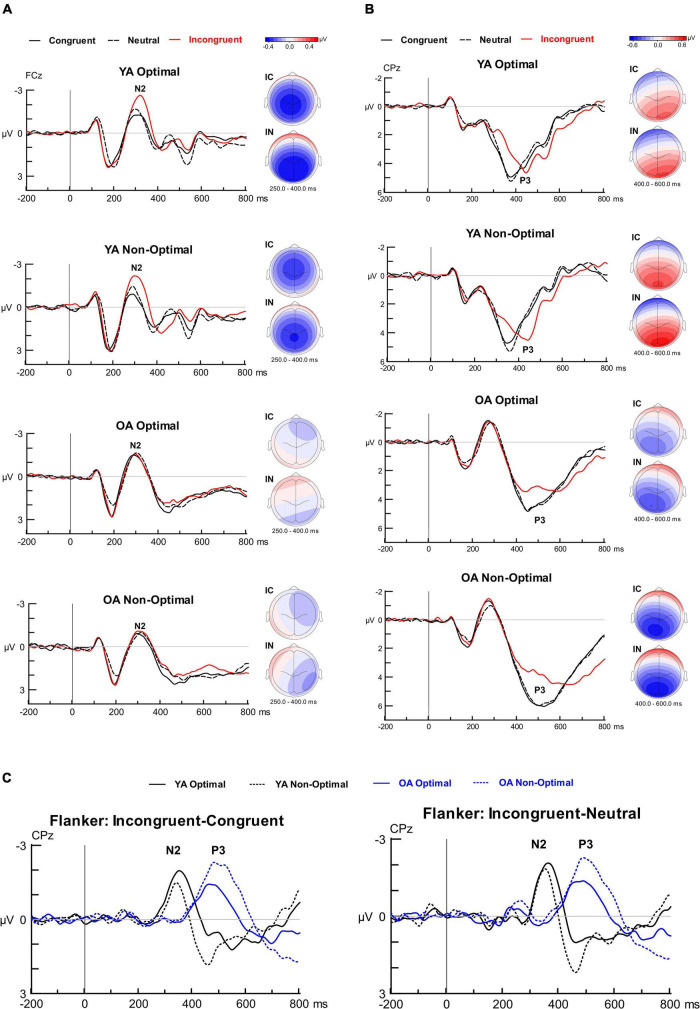
Grand average waveforms, scalp topographies and difference waveforms for the Flanker task. **(A)** Grand-averaged ERPs time-locked to stimulus onset and averaged over congruent, incongruent, and neutral conditions separately for the four groups. The N2 ERP component is depicted at electrode site FCz and topographical iso-contour maps are presented to the right of the grand-averaged ERPs (IC, Incongruent—Congruent; IN, Incongruent—Neutral). **(B)** Grand-averaged ERPs time-locked to stimulus onset and averaged over congruent, incongruent, and neutral conditions separately for the four groups. The P3 ERP component is depicted at electrode site CPz and topographical iso-contour maps are presented to the right of the grand-averaged ERPs (IC, Incongruent—Congruent; IN, Incongruent—Neutral). **(C)** Difference waveforms (Incongruent—Congruent and Incongruent—Neutral) at electrode site CPz depicting the N2 and P3 components for the four groups.

**N2.** Figure 5 displays the Flanker ERP findings. The analysis for the Flanker N2 latencies showed a significant Group by Condition interaction, *F*(2, 198) = 11.83, *p* < 0.001, η*_*p*_*^2^ = 0.107. OAs had longer N2 latencies than YAs for Neutral trials (*p* = 0.028) but not for Congruent (*p* = 0.539) or Incongruent (*p* = 0.205) trials. For YAs, N2 latencies for Incongruent trials were longer than Congruent or Neutral trials (both *p* < 0.001), and did not differ between Congruent and Neutral trials (*p* = 0.997). For OAs, N2 latencies for Neutral were longer than Incongruent (*p* = 0.007) and Congruent (*p* = 0.008) trials, and did not differ between Incongruent and Congruent trials (*p* = 0.998).

The analysis for Flanker N2 mean amplitudes showed a Group by Condition interaction, *F*(2, 198) = 15.96, *p* < 0.001, η*_*p*_^2^* = 0.139. *Post hoc* univariate ANOVAs demonstrated significantly greater N2 amplitude modulation for YAs than OAs for both Incongruent-Congruent and Incongruent-Neutral interference effects (both *p* < 0.001). N2 amplitudes for Incongruent trials were larger for YAs than OAs (*p* = 0.041); amplitudes did not significantly differ between groups for Congruent (*p* = 0.755) or Neutral (*p* = 0.720). No other effects or interactions involving Group, Condition, or TOD reached significance for N2 latency or amplitude. ERP-behavioral correlations with accuracy demonstrated that a greater Incongruent-Congruent N2 modulation in YAs was significantly correlated with a greater Incongruent-Congruent Flanker effect in accuracy (*r* = 0.422, *p* = 0.002), whereas this was not significant in OAs (*r* = 0.061, *p* = 0.566; *z* = 1.92, *p* = 0.028). Neither of these correlations reached significance when conducted with the Incongruent-Neutral effect (YA: *r* = 0.387, *p* = 0.176; OA: *r* = 0.081, *p* = 0.566). Both correlations showed a marginal difference between age groups (*z* = 1.61, *p* = 0.054). As for ERP-behavior correlations with RTs, a greater Incongruent-Neutral N2 modulation in OAs was correlated with a greater Incongruent-Neutral Flanker effect in RTs (*r* = 0.289, *p* = 0.038), whereas this did not reach significance in YAs (*r* = 0.008, *p* = 0.957), with the difference in correlations between age groups reaching marginal significance (*z* = –1.43, *p* = 0.077). Neither of these correlations reached significance when conducted with the Incongruent-Congruent effect (YA: *r* = 0.109, *p* = 0.447; OA: *r* = 0.250, *p* = 0.074), and these correlations did not differ between age groups (*z* = –0.72, *p* = 0.236).

**P3.** For the Flanker P3 latencies, the analysis showed a Group by TOD interaction, *F*(1, 99) = 11.20, *p* = 0.001, η*_*p*_^2^* = 0.102. In YAs, the P3 latencies were longer in the Optimal than Non-Optimal TOD (*p* = 0.008), whereas in OAs the P3 latencies were longer in the Non-Optimal than Optimal TOD (*p* = 0.045). The analysis also showed a main effect of Group, *F*(1, 99) = 85.92, *p* < 0.001, η*_*p*_^2^* = 0.465, with longer P3 latencies in OAs than YAs. Additionally, there was a main effect of Condition, *F*(1.56, 154.17) = 51.14, *p* < 0.001, η*_*p*_*^2^ = 0.341. Pairwise comparisons showed longer P3 latencies for Incongruent than Congruent (*p* < 0.001) or Neutral trials (*p* < 0.001), and no significant difference in latency between Congruent and Neutral trials (*p* = 0.564).

For P3 mean amplitude, the ANOVA yielded a three-way Group by Condition by TOD interaction, *F*(1.53, 151.46) = 7.16, *p* = 0.003, η*_*p*_*^2^ = 0.067. Follow-up two-way ANOVAs showed, in the Non-Optimal TOD, a larger P3 modulation in OAs than YAs for both Incongruent-Congruent and Incongruent-Neutral interference effects (both *p* < 0.001). In the Optimal TOD, there was no significant difference in P3 modulation between age groups for either the Incongruent-Congruent (*p* = 0.426) or Incongruent-Neutral interference effects (*p* = 0.260). For OAs, the P3 for Incongruent trials was smaller than either Congruent or Neutral trials at both Optimal and Non-Optimal testing times (*p* < 0.001 for all). For YAs, the P3 was smaller for Incongruent than Congruent trials (*p* = 0.046) in the Optimal TOD, whereas P3 amplitudes did not significantly differ between conditions in the Non-Optimal TOD. No other effects or interactions involving Group, Condition, or TOD reached significance for P3 latency or amplitude.

ERP-behavior correlations with accuracy demonstrated that, in OAs, the P3 modulation was also significantly correlated with a greater Flanker effect in accuracy (*r* = 0.369, *p* = 0.007 for Incongruent-Congruent, *r* = 0.315, *p* = 0.023 for Incongruent-Neutral). These did not reach significance in YAs (Incongruent-Congruent: *r* = 0.044, *p* = 0.757; Incongruent-Neutral: *r* = 0.057, *p* = 0.690). The difference in correlations between age groups reached significance for Incongruent-Congruent (*z* = –1.69, *p* = 0.045) but not for Incongruent-Neutral (*z* = –1.33, *p* = 0.093). ERP-behavior correlations with RTs demonstrated that, in YAs, a greater P3 modulation was significantly correlated with greater Flanker effect in RTs (*r* = 0.468, *p* = 0.001 for Incongruent-Congruent; *r* = 0.448, *p* = 0.001 for Incongruent-Neutral). These correlations also reached significance in OAs (*r* = 0.501, *p* < 0.001 for Incongruent-Congruent, *r* = 0.375, *p* = 0.006 for Incongruent-Neutral). These correlations did not significantly differ between age groups (Incongruent-Congruent: *z* = –0.21, *p* = 0.416; Incongruent-Neutral: *z* = –0.69, *p* = 0.247).

**Summary.** We observed a significant Group by TOD effect on Flanker P3 latencies. That is, in YAs the P3 latencies were longer during optimal testing times (evening) than non-optimal times (morning), whereas in OAs, P3 latencies were longer during non-optimal (evening) than optimal times (morning). OAs also demonstrated longer latencies for Flanker N2 for Neutral trials only and delayed P3 latencies for all task conditions compared to YAs. With regards to mean amplitude, YAs showed greater Flanker N2 amplitude modulations than OAs, with a greater Flanker N2 correlating with poorer inhibition performance (i.e., greater Flanker effect). Notably, OAs demonstrated a greater Flanker P3 amplitude modulation than YAs, but only during non-optimal testing times, and not for optimal testing times. This Flanker P3 modulation indexed performance in interference control in both age groups.

## Discussion

The present study investigated how age and TOD might interact during tasks engaging interference control and response inhibition using behavioral and electrophysiological measures. Electrophysiological but not behavioral findings demonstrated TOD differences in YAs and OAs. More specifically, a synchrony effect was found in ERP indices of response inhibition, where TOD modulated the neural processing of response inhibition similarly across groups. For interference control, differential TOD effects were found between groups, with increased inhibitory processing demands displayed by OAs during the non-optimal TOD.

### Response Inhibition

Behavioral measures of Go-NoGo task performance demonstrated group differences in Go RTs, with OAs displaying slower RTs than YAs. However, contrary to our predictions, no differences in accuracy were found between the two age groups. Ceiling effects on Go trials limit the extent to which we can draw firm conclusions about the role of age and TOD on inhibition performance. The slower RTs suggest that OAs were more cautious in responding on Go trials to avoid errors on no-go trials. These behavioral results mirror those of Vallesi (2011) who showed slower go responses by OAs but comparable accuracy to YA controls on a Go-NoGo task. The absence of an age-related deficit in inhibitory performance has also been demonstrated by Kardos et al. (2020) who actually found that OAs made fewer commission errors on the Go-NoGo task than YAs, but this was coupled with slower RTs, suggesting a speed-accuracy trade-off.

ERP findings from the Go-NoGo task in the current study are in line with prior electrophysiological research showing age-related deficits in neural processing speed and response inhibition, as reflected by age-related differences in N2 and P3 latency and mean amplitude (Falkenstein et al., 2002; Vallesi and Stuss, 2010; Vallesi, 2011; Lucci et al., 2013; Staub et al., 2014). The present data confirm the age-related slowing of N2 and P3 latency previously reported in past studies and are consistent with the general view that N2 and P3 latency are indices of information processing speed (Polich, 1991; Kutas et al., 1994; McEvoy et al., 2001; Lorenzo-López et al., 2008; Saliasi et al., 2013). Although OAs were able to withhold their behavioral responses with a comparable accuracy to YAs, differential alterations in neurophysiological markers related to inhibitory control were observed between YAs and OAs. A greater N2 modulation was found in YAs than OAs, and a greater P3 modulation was found in OAs than YAs. The larger N2 modulation among YAs suggests that YA participants engage more inhibitory resources during the early stage of inhibitory processing and attempt to monitor and resolve conflict with an emphasis on speed over accuracy, differentiating between Go and NoGo stimuli as early as the time of N2 (i.e., approximately 200–400 ms) (Paxton et al., 2008; Williams et al., 2016). OAs, in contrast, recruit a similar amount of resources to evaluate target stimuli regardless of trial type (Go vs. NoGo).

Our results are also consistent with previous ERP aging Go-NoGo studies showing that the P3 component is more affected by aging than the N2 (e.g., Vallesi and Stuss, 2010; Vallesi et al., 2010; Lucci et al., 2013; Staub et al., 2014), as reflected by an enhanced P3 amplitude difference between No-Go trials and Go trials in OAs. This amplitude difference suggests that OAs may use different strategies with respect to YAs to perform the task, both in the case of conflict monitoring and action execution/suppression. This change may aid in performance by preventing false alarms, allowing OAs to perform at a similar level to YAs; however, the execution speed is reduced, as could be expected when more controlled processing is adopted. Taken together, these data suggest that inhibitory control is a multistage process, in which aging influences the later stages, as indexed by the P3 modulation, more so than the earlier stages, as indexed by the N2 modulation.

In addition to age-related differences in response inhibition, TOD differences in response inhibition were also apparent in ERPs. Both YAs and OAs showed a greater modulation of N2 and greater P3 amplitude during the non-optimal than optimal TOD, demonstrating a synchrony effect in the neural processing of response inhibition and compensatory attention, respectively. The brain-behavior analyses confirmed that greater P3 modulations between NoGo and Go trials were associated with poorer behavioral performance (i.e., greater difference in accuracy between Go and NoGo conditions) in YAs. Furthermore, TOD-related alterations in neurophysiological markers related to inhibitory control were observed in both early (N2 amplitude) and later (P3 amplitude) stages of processing in YAs and OAs. These findings taken together indicate that the observed TOD differences in ERPs arise from circadian-related deficits in inhibition.

### Interference Control

In the current study, behavioral performance on the Flanker task suggested age-related strategy differences in processing irrelevant information. Relative to YAs, OAs exhibited a decreased interference effect in accuracy and an increased interference effect in RT, suggestive of a speed-accuracy trade-off. These findings fit with others showing that OAs prioritize accuracy over speed (Salthouse, 1979; Dirnberger et al., 2010; Starns and Ratcliff, 2010; Forstmann et al., 2011; Maillet et al., 2020), including studies examining Flanker performance in healthy aging (Wild-Wall et al., 2008; Hsieh and Fang, 2012; Hsieh and Lin, 2014). Lack of TOD effects on Flanker performance may be attributed to ceiling effects on Congruent and Neutral trials. As for ERPs, YAs demonstrated an overall larger N2 modulation in amplitude than OAs across TOD. In support of our predictions, OAs demonstrated a larger P3 amplitude modulation than YAs, but only for the non-optimal TOD. In the present study, we showed a negative relationship between the N2 and P3 modulation and performance, such that larger ERP indices of response inhibition were associated with poorer behavioral performance in both YAs and OAs. More specifically, the speed-accuracy trade-off between younger and older adults was further demonstrated in the brain-behavior analyses showing that greater N2 modulations between incongruent and control trials were associated with poorer accuracy in YAs and greater RT Flanker effects in OAs (as evidenced by greater differences in accuracy and reaction time between Incongruent and control conditions). Consistent with prior ERP research examining Flanker performance in aging, the reduced N2 modulation in OAs suggests an emphasis on performance accuracy by intensifying central target processing to reduce flanker interference (Wild-Wall et al., 2008; Hsieh and Fang, 2012; Hsieh et al., 2012). Furthermore, OAs may be resolving the conflicting information in the Flanker task by adopting a strategic, top-down enhancement of visual processing of the central targets during the initial stages of inhibitory processing.

Interestingly, the finding that OAs only demonstrated a larger P3 modulation than YAs during their non-optimal TOD suggests that OAs continue to benefit from their strategy choices during the later stages of inhibitory processing involving interference resolution so long as they completed the task during their optimal TOD. Brain-behavior analyses showed that greater P3 modulation was associated with a greater Flanker effect in RT (i.e., poorer inhibition) for YAs. Brain-behavior analyses further explained enhanced P3 modulations among OAs. This is in line with previous research suggesting that age differences tend to be exaggerated when testing is done during non-optimal times of day (Hasher et al., 2005). In fact, circadian typology and TOD have been suggested to be so influential that age-related impairments on cognitive tasks may even be undetectable when OAs are assessed in the morning, or optimal TOD (Schmidt et al., 2007). Present findings bear resemblance to other studies showing age-related impairments only reaching statistical significance when comparing between YAs and OAs tested in the evening (Intons-Peterson et al., 1998; Hasher et al., 2005). In particular, fMRI research by Anderson et al. (2014) demonstrated that OAs tested at their optimal TOD (morning) relied on a similar set of neural regions underlying inhibitory control in YAs, whereas OAs tested in the afternoon relied on different neural regions. Taken together, our findings suggest that increased circadian-related inhibition demands during the non-optimal TOD affect the later stages of neural processing of inhibitory control in OAs.

Similar to the Go-NoGo task, Flanker P3 peak latencies were more delayed for OAs than YAs. The synchrony effect for interference control also varied across age groups. Within the group of OAs, those tested at non-optimal TOD had longer P3 peak latencies and a greater P3 amplitude modulation compared with those tested at optimal TOD. Surprisingly, this pattern was reversed in YAs, where those tested during non-optimal times of day had earlier P3 peak latencies and a smaller P3 amplitude modulation compared with those tested at optimal times of day. In other words, the smallest P3 amplitude modulation and earliest P3 latencies between the four groups was for YAs tested during non-optimal TOD. Given the lack of ERP research investigating TOD effects, this unexpected finding cannot be interpreted relative to past findings, and warrants future research. We speculate that these differential findings in P3 latency reflect a compensatory response, where YAs may overcompensate for TOD differences to reach comparable performance.

### Theoretical Implications of Aging and Time of Day Effects on Inhibitory Control

Results from the present study have important theoretical implications for the neural correlates underlying inhibitory control in healthy aging. YAs showed greater N2 modulation than OAs on both inhibition tasks, and OAs showed greater P3 modulation than YAs on the Go-NoGo task and at non-optimal testing times on the Flanker task. Prior research has shown that across Go-NoGo and Flanker tasks, the N2 reflects conflict monitoring and detection (Nieuwenhuis et al., 2003; Donkers and van Boxtel, 2004), and the P3 differentially reflects response inhibition in the Go-NoGo task and interference resolution in the Flanker task (Band and Van Boxtel, 1999; Kok et al., 2004; Kan et al., 2021). Furthermore, the differential modulation of N2 and P3 waves in OAs and YAs in our study was interpreted as reflecting age-related differences in the stages of inhibitory processing. More specifically, YAs may be more efficient at monitoring and detecting conflict during the early stages of inhibitory processing and OAs during the later inhibitory processing stages involving overt response inhibition and conflict resolution of distracting flankers.

Future research is needed to clarify N2 and P3 modulation differences in YAs and OAs, as some prior aging research examining inhibitory performance has failed to evaluate both ERP components within the same study (e.g., Hsieh and Fang (2012) did not consider P3 amplitude and Vallesi (2011) did not consider N2 amplitude).

These results provide evidence for age-related differences in the use of proactive and reactive control as proposed by the dual-mechanisms of cognitive control framework (Braver et al., 2007; Braver, 2012). According to the framework, proactive control is considered an *early selection* mechanism that works to maintain goal-relevant information, biasing attention to optimally respond to task demands. In contrast, reactive control is considered a less efficient *late correction* mechanism that is activated when an interference event is detected. Furthermore, the differential N2 and P3 modulation findings found between YAs and OAs in our study are in line with previous research showing that YAs rely on proactive control and OAs rely predominately on reactive control (Braver and West, 2008; Paxton et al., 2008; Czernochowski et al., 2010; Hasher and Campbell, 2020).

A second major theoretical contribution of the present study is that it extends prior behavioral TOD research by demonstrating a substantial impact of circadian misalignment on the neural correlates of inhibitory control. TOD effects were undetectable in behavioral measures despite clear TOD differences in ERP measures, suggesting that both YAs and OAs may be relying on certain strategies in order to perform at comparable levels across times of day. In line with our findings, Smit et al. (2020) examined TOD effects in YAs using ERPs and showed TOD differences in several ERP components of visual distraction. Similarly, no TOD differences were found in behavioral measures, further highlighting the sensitivity of electrophysiological measures in capturing TOD differences. While ceiling effects on behavioral measures limit our conclusions about the behavioral results, we specifically chose a simplified task design in order to isolate the underlying neural processes involved in YAs’ and OAs’ inhibitory performance as a function of TOD (Moreno et al., 2014). While ERP measures in our study demonstrated differences that behavioral measures were not sensitive enough to detect, it is quite possible that with a more complex taxing inhibition task (e.g., reducing the response time window or increasing similarity between distractors and the target stimulus), behavioral differences in TOD may become apparent.

The ERP findings from the present study demonstrated a synchrony effect in Go-NoGo N2 and P3 (i.e., greater N2 modulation and P3 amplitude during the non-optimal relative to optimal TOD) regardless of age group. Consistent with prior TOD research, these results suggest that synchrony effects clearly influence attentional mechanisms and response inhibition with individuals performing more efficiently on inhibition tasks during their optimal TOD. While not all cognitive processes are equally susceptible to TOD effects, in line with our findings, literature on the synchrony effect has shown that tasks involving executive/inhibitory processes associated with prefrontal cortex activity are particularly vulnerable to the influence of circadian misalignment (May and Hasher, 1998; Borella et al., 2010; Anderson et al., 2014).

Flanker ERP findings demonstrated a greater P3 modulation in OAs relative to YAs only during the non-optimal TOD, importantly demonstrating that OAs experience greater demands on inhibitory control resources during periods of circadian mismatch while exhibiting more similar inhibitory processing to YAs during periods of circadian alignment. The present ERP results also clarify the existing interference control literature using Flanker tasks. Despite the vast literature demonstrating greater susceptibility to Flanker interference in OAs than YAs (Zeef and Kok, 1993; Zeef et al., 1996; Colcombe et al., 2005), several studies have failed to find significant behavioral differences (Gunter et al., 1996; Madden and Gottlob, 1997; Falkenstein et al., 2001; Nieuwenhuis et al., 2002; Fernandez-Duque and Black, 2006; Jennings et al., 2007). In fact, a few studies have found that OAs exhibited better interference control performance than YAs (Madden and Gottlob, 1997; Wild-Wall et al., 2008). Our ERP findings suggest that age-related differences in Flanker performance may become obscured or exaggerated if TOD is not accounted for.

A final theoretical contribution of the current study relates to the effect of circadian variation on different inhibitory control subtypes: response inhibition and interference control. ERP findings demonstrated a significant interaction between age and TOD on interference control, but not response inhibition. The differential effects of TOD on inhibitory control subtypes have been demonstrated by previous research. Borella et al. (2010) found that OAs tested in the afternoon but not the morning showed higher Stroop interference effects than YAs, but no such interactions between age and TOD were found in a secondary inhibition task assessing negative priming. Additionally, prior research demonstrated that interference control and response inhibition dissociate in an ERP Go/Nogo Flanker task, finding that the incongruent flanker condition elicited a more centrally distributed topography with a later N2 peak (neural index of inhibitory processing) than the NoGo condition (Brydges et al., 2012).

Tasks of interference control and response inhibition measure distinct constructs of inhibitory control and thus preferentially rely on different brain regions, with greater activation of dorsolateral and ventrolateral regions of the prefrontal cortex shown for response inhibition, and greater activation of the anterior cingulate cortex for interference control (Blasi et al., 2006). Furthermore, the discrepant pattern of TOD results reported for our Flanker and Go-NoGo tasks may be accounted for by the fact that these effects reflect different inhibitory functions, which maybe differentially sensitive to circadian fluctuations (Hasher et al., 2007). The differential TOD effects emphasize the need to consider inhibitory control as a multifaceted construct comprising several similar yet distinct processes (Nigg, 2000; Friedman and Miyake, 2004) or operations at different points in the flow of information (Hasher and Campbell, 2020).

### Implications for Aging and Time of Day Research in Inhibitory Control

Practical implications include consideration of peak-time during testing to optimize the performance of OAs and YAs on tasks that involve inhibitory control and to achieve a more accurate representation of the magnitude of age-related inhibition differences reported in the literature. Given the accumulation of behavioral evidence, and now neurophysiological evidence from our study, showing that TOD can modulate age-related differences in interference control processing/efficiency, information regarding circadian typology and TOD should be reported or controlled routinely. As emphasized by Hasher et al. (2005, 2007), failing to take TOD and chronotype into account may minimize or exaggerate age-related differences in inhibition performance and potentially result in misinterpretations regarding age-related cognitive decline (particularly when all OA participants are tested in the late afternoon/evening hours). For instance, May and Hasher (1998) reported TOD modulations in a Trail Making Test, a common measure of executive functioning included in neuropsychological assessments. Additionally, the increased inhibition demands during testing at non-optimal times of day are further exacerbated for OAs with cognitive impairment relative to healthy OAs (Paradee et al., 2005; Rowe et al., 2021). For these reasons, TOD effects should be considered in routine clinical practice and in research studies examining executive functions such as inhibitory control to avoid misinterpretation of results during improperly timed cognitive assessments.

### Limitations and Future Directions

Chronotype was operationalized in our study using the MEQ, which is a subjective self-reported measure. While there is good current evidence for the validity of the MEQ (i.e., as a correlate with physiological measures of circadian phase, Horne and Östberg, 1976; Roenneberg et al., 2003), future research should consider including objective chronotype metrics such as actigraphy. Another limitation of our study was that YAs had slightly higher anxiety and depression scores on the HADS relative to OAs. However, there is evidence that the HADS anxiety scale overestimates the extent of clinical anxiety in student populations like the one studied here (Andrews et al., 2006). Furthermore, the present study used a forward high-pass filter to improve the signal-to-noise ratio of the ERPs from older adults, who are more prone to head movements that introduce low-frequency artifacts. Our choice of high-pass filter settings may have influenced later stage ERP components and reduced between-group differences in ERP measures (Tanner et al., 2015). To address this issue, future ERP research should examine TOD differences in healthy aging using non-causal high-pass filtering techniques. Finally, the strict inclusion and exclusion criteria of OAs in the present study limits the generalizability of our findings. That is, the present sample of OAs was quite healthy, potentially minimizing differences in behavioral and neural functioning between YAs and OAs that would otherwise be present in the general population of OAs with typical age-related pathologies. Nonetheless, our strict inclusion/exclusion criteria allowed us to confirm that the age-related differences in inhibitory control demonstrated in our study were not due to an underlying pathology, like mild cognitive impairment, which is characterized by deficits in inhibitory control (Rabi et al., 2020).

Despite the aforementioned limitations, findings from this study can be used as a potential springboard for broader examinations of the complex relationship between aging, TOD, and executive functioning using electrophysiological measures. Not only do our findings advance knowledge of underlying changes in the neural mechanisms of inhibitory control associated with TOD and aging, they also have future applications in studying TOD influences in populations with known inhibitory control deficits. For example, this could include individuals with mild cognitive impairment, Alzheimer’s disease, Parkinson’s disease, attention-deficit hyperactivity disorder, autism, and depression (Schachar et al., 1993; Amieva et al., 2004; Palmwood et al., 2017; Schmitt et al., 2018; Martyr et al., 2019; Rabi et al., 2020; Chow et al., 2021).

Future research would also benefit from assessing TOD influences on the neurophysiological correlates of inhibitory control using a broader range of inhibition tasks to confirm TOD differences in inhibitory control subtypes and ensure that effects are subtype-specific rather than just task-specific. To clarify further the relationship between TOD and aging on inhibitory processing, future ERP research should also consider whether manipulating inhibitory task demands modulates cognitive aging and TOD effects. For example, Vallesi et al. (2010) demonstrated that OAs’ compensatory brain responses engaged the more extensive frontoparietal brain network to overcome a prepotent and inappropriate response only when the task was more complex, suggesting the need to further investigate task complexity in relation to the effects of aging and TOD. Finally, our research has demonstrated TOD and age-related differences in inhibitory processing as reflected by cognitive ERPs, but future research should also examine the role of age and TOD on sensory inhibition. Age-related differences in sensory inhibition have recently been demonstrated using sensory-evoked potentials in multiple sensory domains (Alain et al., 2022), highlighting the need to investigate the age-TOD-inhibition relationship in the context of sensory processing.

## Concluding Remarks

Neurophysiological results from this study showcase the influence of age-related circadian patterns on the inhibitory subtypes of response inhibition and interference control. For response inhibition, ERPs from OAs and YAs demonstrated synchrony effects. Age-related differences in the ERP correlates of interference control were found only during the non-optimal TOD. Our results support the hypothesis of TOD effects on inhibition-related mechanisms and age-related effects of TOD on interference control mechanisms, establishing for the first time that electrophysiological markers can substantially contribute toward elucidating the effects of aging and TOD on inhibitory control. Given the mixed behavioral findings regarding age-related differences in inhibition reported in prior research, particularly for the Flanker task, ERP measures in our study showed both age-related and TOD-related differences in inhibitory processing that behavioral measures were not sensitive enough to detect. These changes that accompany circadian demands in inhibition highlight the importance for future research studies in aging to measure and control for circadian typology.

## Data Availability Statement

The raw data supporting the conclusions of this article will be made available by the authors, without undue reservation.

## Ethics Statement

The studies involving human participants were reviewed and approved by the Research Ethics Board of the Rotman Research Institute at Baycrest Centre. The patients/participants provided their written informed consent to participate in this study.

## Author Contributions

RR, LH, NDA, and CA conceived and designed the experiments. RR, RC, and SP performed the experiments and collected the data. RR, RC, SP, and SG analyzed the data. RR, RC, LH, SG, NDA, and CA interpreted the results of the experiments. RR and RC drafted the manuscript. All authors edited and revised the manuscript and approved the final version of the manuscript.

## Conflict of Interest

The authors declare that the research was conducted in the absence of any commercial or financial relationships that could be construed as a potential conflict of interest.

## Publisher’s Note

All claims expressed in this article are solely those of the authors and do not necessarily represent those of their affiliated organizations, or those of the publisher, the editors and the reviewers. Any product that may be evaluated in this article, or claim that may be made by its manufacturer, is not guaranteed or endorsed by the publisher.

## Footnotes

1 It has been suggested that N2 and P3 latency reflects information processing speed and N2 and P3 amplitudes express inhibitory control functions (Duncan-Johnson and Kopell, 1981; Polich and Herbst, 2000). For this reason, conclusions regarding inhibitory functioning were drawn based on N2 and P3 amplitude results and not latency results.

2 Go RT is considered a measure of processing speed which does not index inhibition. For this reason, Go RT was not included in ERP-behavioral correlations.
