# Supplementary material for: The Effects of Aging and Time of Day on Inhibitory Control: An Event-Related Potential Study
Source: Front Aging Neurosci. 2022 Mar 11;14:821043. doi: 10.3389/fnagi.2022.821043 (PMC8963784; doi:10.3389/fnagi.2022.821043)
Supplement: Supplementary file 1 [file Data_Sheet_1.pdf]

Table 1.

Summary of GLMM Statistics for Behavioral Data, Go-NoGo Task

Effects	Accuracy	Reaction Time
**Omnibus Model**		
Group	X^2 (1) = 1.60, p = .201	F(1, 43329) = 49.94, p < .001
TOD	X^2 (1) = .51, p = .475	F(1, 43329) = .42, p = .516
Group x TOD	X^2 (1) = .18, p = .674	F(1, 43329) = .72, p = .396
Condition	X^2 (1) = 58.00, p < .001	
Group x Condition	X^2 (1) = .57, p = .449	
TOD x Condition	X^2 (1) = 2.12, p = .145	
Group x TOD x Condition	X^2 (1) = .07, p = .788	
**Final Model**		
Group		F(1, 43329) = 50.35, p < .001
Condition	X^2 (1) = 57.46, p < .001	

Note. TOD = time of day. GLMM statistics depict Type III results.

Table 2.

Summary of GLMM Statistics for Behavioral Data, Flanker Task

Effects	Accuracy	Reaction Time
**Omnibus Model**		
Group	X^2 (1) = 2.74, p = .098	F(1, 29121) = 97.63, p < .001
TOD	X^2 (1) = 1.15, p = .284	F(1, 29121) = .06, p = .810
Group x TOD	X^2 (1) = .42, p = .516	F(1, 29121) = .12, p = .734
Condition	X^2 (2) = 58.62, p < .001	F(2, 29121) = 3585.30, p < .001
Group x Condition	X^2 (2) = 7.70, p = .021	F(2, 29121) = 21.90, p < .001
TOD x Condition	X^2 (2) = 2.71, p = .258	F(2, 29121) = .48, p = .619
Group x TOD x Condition	X^2 (2) = 1.62, p = .445	F(2, 29121) = 1.98, p = .1381
**Final Model**		
Group	X^2 (1) = 2.73, p = .099	F(1, 29123) = 99.33, p < .001
Condition	X^2 (2) = 58.32, p < .001	F(2, 29123) = 3583.02, p < .001
Group x Condition	X^2 (2) = 7.68, p = .022	F(2, 29123) = 22.09, p < .001

Note. TOD = time of day. GLMM statistics depict Type III results.

Table 3.

Summary of Statistics of Electrophysiological Data for Go-NoGo and Flanker Tasks.

Measure	Effect	F-statistics	B_{10}	Post-hoc	p-value
**Go-NoGo**					
N2 Latency	Group*	$F(1, 99) = 19.49, p < .001, \eta_p^2 = .165$	720.60	OA > YA*	<.001
	TOD	$F(1, 99) < .00\ p = .962, \eta_p^2 < .001$	.33		
	Condition	$F(1, 99) = .44, p = .511, \eta_p^2 = .004$	.15		
	Group x TOD	$F(1, 99) = .12, p = .728, \eta_p^2 = .001$	.40		
	Group x Condition	$F(1, 99) = 3.77, p = .055, \eta_p^2 = .037$	.55		
	TOD x Condition	$F(1, 99) = .25, p = .620, \eta_p^2 = .002$	.20		
	Group x TOD x Condition	$F(1, 99) = .74, p = .391, \eta_p^2 = .007$	.32		
N2 Amplitude	Group	$F(1, 99) = 8.36, p = .005, \eta_p^2 = .078$	8.68	-	
	TOD	$F(1, 99) = .14, p = .906, \eta_p^2 < .001$	.34		
	Condition	$F(1, 99) = 7.71, p = .007, \eta_p^2 = .072$	2.91	-	
	Group x TOD	$F(1, 99) = 1.14, p = .289, \eta_p^2 = .011$	.56		
	Group x Condition*	$F(1, 99) = 12.45, p = .001, \eta_p^2 = .112$	33.37	OA: NoGo = Go	.594
				YA: NoGo > Go*	<.001
				Go: OA = YA	.121
				NoGo: YA > OA*	<.001
	TOD x Condition*	$F(1, 99) = 6.96, p = .010, \eta_p^2 = .066$	3.44	Opt: NoGo = Go	.923
				NOpt: NoGo > Go*	<.001
				Go: Opt = NOpt	.378
				NoGo: Opt = NOpt	.329
	Group x TOD x Condition	$F(1, 99) = .94, p = .336, \eta_p^2 = .009$	.44		
P3 Latency	Group*	$F(1, 99) = 14.54, p < .001, \eta_p^2 = .128$	120.50	OA > YA*	<.001
	TOD	$F(1, 99) < .00, p = .986, \eta_p^2 < .001$	.26		
	Condition*	$F(1, 99) = 10.98, p = .001, \eta_p^2 = .100$	18.76	Go > NoGo*	.001
	Group x TOD	$F(1, 99) = .13, p = .716, \eta_p^2 = .001$	.39		
	Group x Condition	$F(1, 99) = 2.56, p = .113, \eta_p^2 = .025$	.62		
	TOD x Condition	$F(1, 99) = .25, p = .619, \eta_p^2 = .003$	.27		
	Group x TOD x Condition	$F(1, 99) = .09, p = .772, \eta_p^2 = .001$	.28		

P3 Amplitude	Group	$F(1, 99) = 1.77, p = .186, \eta_p^2 = .018$	.60		
	TOD	$F(1, 99) = 4.37, p = .039, \eta_p^2 = .042$	1.76		
	Condition	$F(1, 99) = 31.92, p < .001, \eta_p^2 = .244$	3.18×10^5	-	
	Group x TOD	$F(1, 99) = .11, p = .741, \eta_p^2 = .001$	.40		
	Group x Condition*	$F(1, 99) = 7.99, p = .006, \eta_p^2 = .075$	4.67	OA: NoGo > Go*	<.001
				YA: NoGo = Go	.050
				Go: OA = YA	.894
				NoGo: OA > YA*	.022
	TOD x Condition	$F(1, 99) = .08, p = .784, \eta_p^2 = .001$	.22		
	Group x TOD x Condition	$F(1, 99) = .71, p = .402, \eta_p^2 = .007$	.30		
**Flanker**					
N2 Latency	Group	$F(1, 99) = .28, p = .600, \eta_p^2 = .003$	.34		
	TOD	$F(1, 99) = .68, p = .412, \eta_p^2 = .007$	.42		
	Condition	$F(2, 198) = 4.94, p = .008, \eta_p^2 = .047$	1.87	-	
	Group x TOD	$F(1, 99) = 2.97, p = .088, \eta_p^2 = .029$	1.09		
	Group x Condition*	$F(2, 198) = 11.83, p < .001, \eta_p^2 = .107$	1286.22	YA: Inc > Con*	<.001
				YA: Inc > Neu*	<.001
				YA: Con = Neu	.997
				OA: Neu > Inc*	.007
				OA: Neu > Con*	.008
				OA: Inc = Con	.998
	TOD x Condition	$F(2, 198) = 2.36, p = .097, \eta_p^2 = .023$	.46		
	Group x TOD x Condition	$F(2, 198) = 1.40, p = .249, \eta_p^2 = .014$	.27		
N2 Amplitude	Group	$F(1, 99) = .53, p = .468, \eta_p^2 = .005$	.52		
	TOD	$F(1, 99) = .48, p = .491, \eta_p^2 = .005$	.52		
	Condition	$F(2, 198) = 15.23, p < .001, \eta_p^2 = .133$	3641.46	-	
	Group x TOD	$F(1, 99) = .04, p = .844, \eta_p^2 < .001$	.55		
	Group x Condition*	$F(2, 198) = 15.96, p < .001, \eta_p^2 = .139$	3.93×10^4		
	Inc-Con*	$F(1, 99) = 28.95, p < .001, \eta_p^2 = .226$	3.20×10^4	YA > OA*	<.001
	Inc-Neu*	$F(1, 99) = 13.121, p < .001, \eta_p^2 = .117$	61.11	YA > OA*	<.001
	TOD x Condition	$F(2, 198) = 1.03, p = .358, \eta_p^2 = .010$	.14		
	Group x TOD x Condition	$F(2, 198) < .00, p = .999, \eta_p^2 < .001$	.07		
P3 Latency	Group*	$F(1, 99) = 85.92, p < .001, \eta_p^2 = .465$	3.18×10^{11}	OA > YA*	<.001

	TOD	$F(1, 99) = .24$, $p = .625$, $\eta_p^2 = .002$	.39		
	Condition	$F(1.56, 154.17) = 51.14$, $p < .001$, $\eta_p^2 = .341$	1.36×10^{16}	-	
	Group x TOD*	$F(1, 99) = 11.20$, $p = .001$, $\eta_p^2 = .102$	23.29	YA: Opt > NOpt*<br>OA: NOpt > Opt*	.008<br>.045
	Group x Condition	$F(1.56, 154.17) = .41$, $p = .611$, $\eta_p^2 = .004$	.09		
	TOD x Condition	$F(1.56, 154.17) = 1.00$, $p = .354$, $\eta_p^2 = .010$	.12		
	Group x TOD x Condition	$F(1.56, 154.17) = .47$, $p = .579$, $\eta_p^2 = .005$	.12		
P3 Amplitude	Group	$F(1, 99) = 4.88$, $p = .030$, $\eta_p^2 = .047$	1.92	-	
	TOD	$F(1, 99) = .23$, $p = .637$, $\eta_p^2 = .002$	.50		
	Condition	$F(1.53, 151.46) = 28.25$, $p < .001$, $\eta_p^2 = .222$	1.90×10^6	-	
	Group x TOD	$F(1, 99) = .69$, $p = .407$, $\eta_p^2 = .007$	.76		
	Group x Condition	$F(1.53, 151.46) = 14.93$, $p < .001$, $\eta_p^2 = .131$	1439.06	-	
	TOD x Condition	$F(1.53, 151.46) = .19$, $p = .765$, $\eta_p^2 = .002$	.08		
	Group x TOD x Condition*	$F(1.53, 151.46) = 7.16$, $p = .003$, $\eta_p^2 = .067$	29.47		
	Inc-Con: Group x TOD*	$F(1, 99) = 10.66$, $p = .002$, $\eta_p^2 = .097$	20.81	Opt: OA = YA<br>NOpt: OA > YA*	.426<br><.001
	Inc-Neu: Group x TOD*	$F(1, 99) = 5.59$, $p = .020$, $\eta_p^2 = .053$	2.67	Opt: OA = YA<br>NOpt: OA > YA*	.260<br><.001

Note. B_{10} = Bayes factor; TOD = Time of day; YA = younger adult; OA = older adult; Con= Congruent; Inc = Incongruent; Neu = Neutral; Opt = Optimal time of day; NOpt = Non-optimal time of day. *$p < .05$

Table 4.

Summary of N2 and P3 Peak Latencies and Mean Amplitudes for Go-NoGo and Flanker Tasks.

Measure	Peak Latency (ms)				Mean Amplitude (µV)			
	YA Optimal	YA Non-Optimal	OA Optimal	OA Non-Optimal	YA Optimal	YA Non-Optimal	OA Optimal	OA Non-Optimal
**Go-NoGo N2**								
Go	287.65 (28.28)	282.48 (27.14)	302.04 (33.06)	305.07 (27.64)	-1.52 (1.32)	-0.92 (1.42)	-0.75 (1.38)	-0.89 (1.07)
NoGo	280.64 (22.48)	281.36 (22.12)	306.89 (35.30)	307.28 (29.10)	-1.78 (1.38)	-1.89 (1.60)	-0.51 (1.40)	-0.98 (1.56)
**Go-NoGo P3**								
Go	448.85 (78.46)	440.21 (77.35)	475.46 (75.39)	478.43 (60.16)	0.96 (1.21)	1.61 (1.43)	1.07 (1.52)	1.66 (1.66)
NoGo	414.13 (57.53)	415.10 (38.37)	463.76 (62.89)	469.26 (51.70)	1.31 (1.25)	2.02 (1.33)	2.16 (1.80)	2.50 (1.40)
**Flanker N2**								
Congruent	290.70 (29.26)	285.05 (31.87)	282.29 (38.15)	301.83 (37.41)	-0.49 (1.52)	-0.22 (1.69)	-0.52 (1.36)	-0.37 (1.16)
Incongruent	302.95 (25.94)	299.11 (36.83)	282.10 (36.21)	303.04 (34.20)	-1.09 (1.52)	-0.95 (1.65)	-0.45 (1.30)	-0.41 (1.28)
Neutral	291.75 (27.31)	285.14 (33.18)	299.58 (28.97)	304.71 (34.35)	-0.68 (1.50)	-0.36 (1.73)	-0.52 (1.36)	-0.31 (1.17)
**Flanker P3**								
Congruent	415.87 (69.44)	385.11 (57.82)	484.40 (44.89)	515.64 (60.71)	2.85 (1.62)	2.50 (1.21)	3.35 (2.01)	3.98 (2.11)
Incongruent	459.18 (57.62)	416.34 (57.35)	519.46 (52.25)	549.11 (77.37)	2.53 (1.82)	2.77 (1.19)	2.85 (1.91)	3.04 (1.91)
Neutral	421.87 (58.05)	372.23 (54.70)	485.73 (44.36)	514.94 (67.45)	2.93 (1.75)	2.68 (1.30)	3.51 (2.09)	4.01 (2.07)

Note: YA = younger adult; OA = older adult. Data are in means (SD).
